# Clinical Manifestations and Outcomes of *Rickettsia australis* Infection: A 15-Year Retrospective Study of Hospitalized Patients

**DOI:** 10.3390/tropicalmed2020019

**Published:** 2017-06-20

**Authors:** Adam Stewart, Mark Armstrong, Stephen Graves, Krispin Hajkowicz

**Affiliations:** 1 Department of Infectious Diseases, Royal Brisbane and Women’s Hospital, Brisbane, QLD 4029, Australia; mark.armstrong@health.qld.gov.au (M.A.); krispin.hajkowicz@health.qld.gov.au (K.H.); 2 Australian Rickettsial Reference Laboratory, Geelong, VIC 3220, Australia; graves.rickettsia@gmail.com; 3 School of Medicine, University of Queensland, Brisbane, QLD 4067, Australia

**Keywords:** tick-borne diseases, rickettsial infections, bacteriology

## Abstract

Queensland tick typhus (QTT; *Rickettsia australis*) is an important cause of community-acquired acute febrile illness in eastern Australia. Cases of QTT were identified retrospectively from 2000 to 2015 at five sites in Northern Brisbane through a pathology database. Those included had a fourfold rise in spotted fever group (SFG)-specific serology, a single SFG-specific serology ≥ 256 or SFG-specific serology ≥ 128 with a clinically consistent illness. Cases were excluded on the basis of clinical unlikelihood of QTT infection. Thirty-six cases were included. Fever was found in 34/36 (94%) patients. Rash occurred in 83% of patients with maculopapular being the dominant morphology (70%). Thrombocytopenia, lymphopenia, and raised transaminases were common and occurred in 58%, 69%, and 89% of patients, respectively. Thirty-one of 36 (86%) patients received antibiotic therapy (usually doxycycline) and the time to correct antibiotic (from admission) ranged from 3 to 120 h (mean 45.5 h). Four of 36 (11%) required intensive care unit (ICU) admission for severe sepsis and end-organ support. There were no deaths. QTT has a wide range of clinical and laboratory features. Early and appropriate antimicrobial therapy is important and may prevent severe disease. Further prospective studies are required to identify factors associated with severe infection and sepsis.

## 1. Introduction

Queensland tick typhus (QTT) is an ill-recognized tick-borne disease that occurs along the eastern coast of Australia [1]. It is believed to be a relatively uncommon infection, although its incidence may be significantly underreported [2]. The causative organism, *Rickettsia australis*, is one of 19 known pathogenic *Rickettsia* species worldwide and one of three spotted fever group (SFG) rickettsial infections that occur in Australia, the other two species being *R. honei* (Flinders Island spotted fever) and *R. honei* sub sp. *marmionii* (Australian spotted fever) [1,2]. Clinical illness is often non-specific and difficult to diagnose, with severity ranging from a mild self-limiting illness to fulminant disease and sepsis, including death [3]. Moreover, recent case reports and series have demonstrated the occurrence of more severe disease and complications, shifting the paradigm from the short-lived, mild flu-like illness that was originally described in the retrospective review by Sexton et al. in 1990 [4,5,6].

## 2. Materials and Methods

Study patients were both adults and children identified retrospectively during the period of January 2000–January 2015 at any of the five sites in Metropolitan North Hospital and Health Service (MNHSS) in Queensland, Australia via the local pathology database. MNHSS services an area from north of the Brisbane River to north of Kilcoy, with the population in this area approaching 900,000 [7]. Medical records as well as pathology and radiology databases were reviewed for each identified case. Demographic, clinical, laboratory, and radiographic data were recorded onto a standardized electronic case report form. Clinical features were considered present only if their presence was documented in the medical record. Confirmed or probable cases of QTT included those with a greater or equal to a fourfold rise in spotted fever group (SFG)-specific serology (*Rickettsia rickettsii* IgG); a single SFG-specific serology ≥ 256; or SFG-specific serology ≥ 128 with a clinically consistent illness. A clinically consistent illness was defined as a presentation typical for QTT, without a more likely diagnosis being apparent, as well as a good clinical response to doxycycline or another appropriate antibiotic. Cases were further excluded if serology results represented an old infection, acute illness thought to be acquired overseas, or a known cause of antibody cross-reactivity to rickettsia-specific antigens (e.g., autoimmune conditions). Old infection was defined as an acute, clinically consistent illness in the recent past receiving successful treatment in patients that had no repeat epidemiological exposures and had presented to hospital for what was deemed an unrelated condition. The indirect microimmunofluorescence assay (IFA) utilizing an SFG-specific *Rickettsia rickettsii* antigen was used in serological testing [8].

## 3. Results

Of the 50 cases identified by serological criteria alone through the pathology database, 36 were included after the exclusion criteria was applied. Two patients’ medical records were destroyed and thus inaccessible. Out of the 12 remaining cases, eight had a clinically inconsistent illness, two were deemed to have an old infection and two had acquired their infection overseas.

### 3.1. Symptoms and Signs

Fever (which included subjective reports) was nearly universal, and found in 34/36 (94%) patients (Table 1). In those with fever, the highest recorded temperatures ranged from 37.7 to 40.5 degrees Celsius (median 39.1). The average length of fever from date of admission was 1.4 days. Rash occurred in the majority of patients (30/36, 83%). Of those with rash, maculopapular morphology dominated in 21/30 (70%); while macular (17%), vesicular (10%), and purpura (3%) made up the remainder. Rash distribution was more commonly widespread and global in 21/30 (70%), with local sites including lower limbs (13%), upper limbs (10%), neck or trunk (7%). This would mainly reflect the site of tick bite/inoculation. Eschar was present in only 6/36 (17%) cases, with the lower limb being the most common site. Of all 36 patients, 17 (47%) reported a known tick bite in their presenting history. Lymphadenopathy was found in 10/36 (28%) of patients, with a cervical location twice as common as inguinal (60% compared to 30%); only 1/10 (10%) had generalized lymphadenopathy. Headache was a common presenting feature and was seen in 24/36 (67%). Generalized arthralgia and myalgia were present in 20/36 (56%) each. Respiratory symptoms were relatively uncommon, with cough and dyspnea identified in 10/36 (28%) and 5/36 (14%), respectively. Abdominal pain or tenderness (identified on history or examination), usually in the right upper quadrant, complicated 10/36 (28%) cases. Confusion and drowsiness were rarely observed on presentation, and each featured in 1/36 (3%) patients.

### 3.2. Laboratory and Radiological Findings

**Figure 1:**
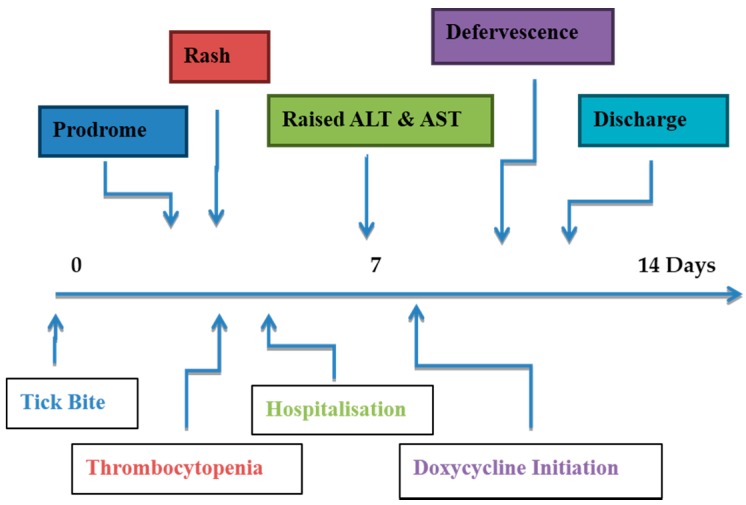
Hypothetical timeline of events in patients with Queensland tick typhus (arrows indicate median values).

Thrombocytopenia (platelet count < 150 × 10^9^/L) was relatively common and occurred in 21/36 (58%) patients at some time during the course of their illness; this was identified more commonly on the day of presentation to hospital (Figure 1). A platelet nadir was typically observed on Day 3–4 of hospitalization, with a minimum value of 13 × 10^9^/L. Reactive thrombocytosis (platelet count > 400 × 10^9^/L) was observed in 12/36 (33%) and was usually seen after Day 7. Lymphopenia (lymphocytes < 1.1 × 10^9^/L) was also seen in the majority of patients (25/36 (69%)) and was more likely to be present on Day 0 and normalized by Day 3 of illness. Elevated serum transaminases (alanine aminotransferase (ALT) > 45 IU/L or aspartate aminotransferase (AST) > 41 IU/L) were observed in 32/36 (89%) patients and usually reached a maximum at Day 4–6, with a maximum ALT value of 833 IU/L and a maximum AST value of 944 IU/L. Renal impairment was uncommon, occurring in 5/36 (14%) and was usually prerenal as a result of hypovolemia secondary to sepsis. Significant non-nephrotic range proteinuria complicated a further 3/36 (8%) cases. C-reactive protein (CRP) was frequently raised between 100 and 250 mg/L on Day 0, with a precipitous decline thereafter, following the institution of doxycycline therapy. Coagulopathy was rare and complicated 3/36 (8%) cases, typically in the setting for severe infection and sepsis. Chest X-ray changes were observed on presentation in 7/36 (19%) patients and mainly included small bilateral pleural effusions and/or basal consolidation. Hepatomegaly was the most common abnormality identified on abdominal imaging and occurred in 6/36 (17%) patients.

### 3.3. Treatment and Course of Illness

Thirty-one of 36 (86%) patients received antibiotic therapy for QTT. Of those treated, 29/31 (94%) were treated with doxycycline and 2/31 (6%) with azithromycin. The duration of antibiotic treatment ranged from three to 14 days (most commonly 7–10 days). Doxycycline was usually dosed at 100 mg orally twice daily (90%), alternatively at 100 mg once daily (10%). Only 4/29 (14%) received a loading dose of 200 mg orally. Where azithromycin was used, 250 mg orally daily and 500 mg orally daily represented each of the cases, for five and three days respectively. The time to correct antibiotic therapy (measured from the time presented to the emergency department) ranged from 3 to 120 h (mean 45.5 h). Doxycycline was well-tolerated, with only 5/29 (17%) reporting side effects that included nausea, vomiting, and itch. Hypotension (systolic blood pressure < 90 mm Hg) was common during hospitalization and 12/36 (33%) patients required intravenous fluids to correct this. The duration of hospital admission ranged from one to 60 days (median 6 days). Twenty-seven of 36 (75%) patients were managed entirely on the hospital ward, 5/36 (14%) were sent home for outpatient follow-up, and 4/36 (11%) required intensive care unit (ICU) admission. Long-term complications and sequelae were not observed. 

### 3.4. Severe Outcomes and Sepsis

Severe sepsis and the requirement of organ support was seen in 4/36 (11%) patients. Of the severe cases, patient age ranged from 17 to 63 years; the male-to-female ratio was 1:3. The length of hospital stay ranged from nine to 60 days. Three of four (75%) lived within a five-kilometer radius of each other and presented in March-April. All four patients admitted to the ICU had small bilateral pulmonary infiltrates, pleural effusions, and respiratory impairment. Two of four (50%) were complicated by acute renal failure and proteinuria. Acidosis and disseminated intravascular coagulation (DIC) complicated 2/4 (50%). Diffuse enteritis/colitis, myositis, a raised troponin, and digital gangrene occurred in one patient for each complication. There were no deaths observed.

## 4. Discussion

QTT has the potential to cause severe disease and organ-specific complications requiring prolonged hospitalization and intensive care support [3]. However, the majority of individuals who acquire this infection and receive appropriate and timely antimicrobial therapy have a short disease course without sequalae [9]. Moreover, this study only evaluated hospitalized patients and likely only sampled severe infections from a larger pool of mild clinical and sub-clinical disease [2]. This group is difficult to quantify, as a diagnosis of any rickettsial infection does not require compulsory reporting in Australia, and serological testing is not performed routinely in the community setting [1,2].

Laboratory and clinical manifestations of SFG infections in Australia were evaluated by Sexton et al. (1991) and provide the basis for our current understanding of QTT [4]. It was likely that *Rickettsia honei* (Flinders Island spotted fever) and *R. honei* sub sp. *marmionii* (Australian spotted fever) were included in the 62 cases retrospectively identified by serology in that study [10]. There has been difficulty in characterizing and defining SFG rickettsial infections in Australia as they share similar clinical manifestations, as well as sharing some overlapping geographical distributions [11]. Although that study reported one fatality from QTT, it labeled the infection as a mild, self-limiting febrile illness with little propensity to cause severe disease; however, outcomes and complications were not specifically addressed.

*R. australis* infection more likely produces a spectrum of illness from mild flu-like symptoms to sepsis and multi-organ failure. Rash morphology findings in our study largely mimicked those of previous results for QTT, with 83% maculopapular or macular and 10% vesicular. This likely represents a generalizable manifestation of all SFG infections in Australia [2]. Accurate rash description among physicians is not universal, and “maculopapular” is often used as a default and was likely over-reported. Although a history of tick bite was consistent with previous reports (47% versus 58%), there were far fewer documented eschars in our study (17% versus 50%). This may imply that *R. australis* endemic to Southeast Queensland has less propensity to cause local skin vasculitis and vessel thrombosis; or that eschar is seen less commonly in severe infection and may represent an intensive immune response that functions to prevent the blood-borne spread of rickettsia [12]. Moreover, the vast difference in the presence of tender lymphadenopathy (28% versus 70%) could represent different illnesses or organisms in previous studies, or differences in examination practices amongst medical practitioners. Thrombocytopenia, lymphopenia, and raised serum transaminases all occur in the majority of patients and are consistent in QTT [8]. Doxycycline is an uncommon cause of deranged liver function tests and can confound this marker of QTT infection during hospitalization [9]. Renal impairment and/or proteinuria is rare in QTT in the absence of severe sepsis, and if present suggests an alternate diagnosis [2]. Unlike Rocky Mountain spotted fever (*R. rickettsii*), which is a known cause of interstitial nephritis, QTT does not have a direct pathogenic effect on the kidney [13]. Patients treated early with appropriate antimicrobial therapy had a rapid clinical improvement that was evident by an improvement in both clinical parameters and markers of acute inflammation (i.e., CRP). 

QTT as a cause of severe sepsis is a novel problem that may be preventable. Eleven percent of study patients required ICU admission and organ support that was associated with organ-specific and general inpatient complications, as well as a prolonged hospital stay. Sepsis in QTT has not been adequately described in the literature. Although numbers were low, severe sepsis had a female predominance of 3:1, but failed to show any association with the presence of comorbidities or age of the patient. Possible factors increasing the risk of ICU admission include low blood pressure, increased heart rate, decreased platelets, and hyperbilirubinemia, need further assessment in larger cohorts of patients with QTT. Interestingly, 3/4 (75%) patients admitted to the ICU lived within a five-kilometer radius of each other (Samford Valley) and presented during the March/April period. This could represent an area of hyperendemicity with multiple tick bites and greater inoculum size as potential contributors to severe disease. A hypervirulent strain of *R. australis* is another possibility. It is known that the mean time to appropriate antibiotics and delayed presentations are the main determinants of outcome in SFG rickettsial disease, especially Rocky Mountain spotted fever (*R. rickettsii*) [13]. 

Despite the potential for multi-organ failure, death has only been documented once in the literature and did not occur in this study [14]. Long-term complications are just as uncommon, with the majority of patients returning to full health post-infection [2,9]. Moreover, there is no evidence of QTT causing chronic infection [9]. No study patients had a documented re-infection. This was despite no lifestyle or occupational changes, or a change in location of residency. This could represent possible protective immunity against *R. australis*, although due to the low incidence of disease, it is difficult to make this conclusion. Cases of prolonged lethargy have been documented infrequently in QTT; it was recorded in 2/36 (5%) of study patients. The link between rickettsial infection and chronic fatigue syndrome is yet to be confirmed [9].

## 5. Conclusions

QTT is an Australian SFG rickettsial infection with a growing public health importance. It is an important cause of community-acquired febrile illness with a wide spectrum of clinical and laboratory manifestations that need to be recognized promptly in order to institute timely antimicrobial therapy. An accurate delineation of the geographical distribution of disease, including areas of hyperendemicity, could further assist in the identification of these patients. Additional key risk factors for severe sepsis in QTT could provide insight into potential public health procedures for prevention. This could offer significant benefit to patients and the community by reducing hospitalizations and ICU admissions for QTT. Further prospective studies are required to refine current knowledge and ultimately impact clinical practice. 

## Figures and Tables

**Table 1:** Key clinical and laboratory characteristics before or during admission in 36 patients with Queensland tick typhus (QTT).

Characteristic	Number of Patients (%)
Fever	34/36 (94%)
Rash	30/36 (83%)
Macular or maculopapular	26/36 (72%)
Vesicular or other	4/36 (11%)
Myalgia	20/36 (56%)
Headache	24/36 (67%)
Abdominal pain	10/36 (28%)
Lymphadenopathy	10/36 (28%)
Eschar	6/36 (17%)
Thrombocytopenia	21/36 (58%)
Elevated aminotransferases	32/36 (89%)
Lymphopenia	25/36 (69%)
